# Karyotypic variation of two populations of the small freshwater stingray *Potamotrygon wallacei* Carvalho, Rosa & Araújo 2016: A classical and molecular approach

**DOI:** 10.1371/journal.pone.0278828

**Published:** 2023-01-20

**Authors:** Alex M. V. Ferreira, Patrik F. Viana, Leandro Marajó, Eliana Feldberg

**Affiliations:** 1 Programa de Pós-Graduação em Genética Conservação e Biologia Evolutiva – PPG GCBEv, Instituto Nacional de Pesquisas da Amazônia – INPA, Manaus, Amazonas, Brazil; 2 Laboratório de Genética Animal, Coordenação de Biodiversidade, Instituto Nacional de Pesquisas da Amazônia – INPA, Manaus, Amazonas, Brazil; Sichuan Agricultural University at Chengdu, CHINA

## Abstract

Potamotrygoninae comprises a group of Neotropical fishes with an ancient relationship with marine environments. In the last few years, 11 new *Potamotrygon* species were described, including *Potamotrygon wallacei* Carvalho, Araújo e Rosa 2016. Cytogenetic data about this species are limited to classical markers (Giemsa, C-Banding and Ag-NOR techniques), these studies highlighted a rare sexual chromosome system XX/X0 with males presenting 67 chromosomes and females 68 chromosomes. The classical analyses performed here reveled populational variation in the karyotype formula, as well as, in the heterochromatin regions. Besides the classical markers, our molecular experiments showed multiple sites for 18S rDNA sequence (including in the X chromosomes) and single sites for 5S rDNA sequence, we did not find interstitial telomeric sequences. In addition, (AC)_15_, (AG)_15_, and (CAC)_15_ microsatellites showed association with the several autosome pair, and the (GT)_15_ clutters were found in only one population. On the other hand, (GATA)_4_ sequence showed association with the sexual chromosomes X in all males and females analyzed. Our results showed that pericentric inversions, in addition to fusions, shaped the karyotype of *P*. *wallacei* once we found two populations with distinct karyotype formula and this could be a result of the past events recovered by our modeling experiments. Besides, here we described the association of 18S and (GATA)_4_ motifs with sexual chromosomes, which indicated that these sequences had a novel in the differentiation of sexual chromosomes in *P*. *wallacei*.

## Introduction

The subfamily Potamotrygoninae is a group of South American stingray lineages of marine origin that comprises over 40 species restricted to freshwater environments [1–7]. Currently, four genera, *Heliotrygon*, *Plesiotrygon*, *Paratrygon*, and *Potamotrygon* are recognized for this subfamily [8, 9], occurring in different South America river basins that flow into the Caribbean and Atlantic Sea [2, 10]. In the Amazon basin, these stingrays are found in different types of water, such as black water, clear water and white water [2]. Additionally, some physiological and ecological traits, such as their low fecundity, late maturation and small home range size increase the vulnerability to indiscriminate fishing [11–14].

In the past years, some studies have revealed a hitherto unknown diversity into the Potamotrygoninae subfamily, with the description of a new genus and several new species [7, 8, 15]. The genus *Potamotrygon*, for instance, is the most speciose among all potamotrygonins, with 11 new described species only in the past decade [8, 16–20], including *Potamotrygon wallacei* Carvalho, Araújo e Rosa 2016 [21], one of the most iconic freshwater stingray species in the Amazon.

*Potamotrygon wallacei* is the smallest stingray of the *Potamotrygon* genus, and although it has been known since the Alfred Russel Wallace’s journey into the Amazon, the species was only named (in honor of Wallace) in 2016 [13, 21]. *P*. *wallacei* is an endemic species, with distribution apparently restricted to the Rio Negro basin [21], however, the real extent of its distribution as well as many aspects of its natural history remains unknown. Due to its small size, *Potamotrygon wallacei* is a very appreciated species in the aquarist market, and although it is not even listed in the IUCN, this species has been legally and illegally exported to supply the international market even before its formal description [11, 13, 21].

Cytogenetic data about this species revealed the presence of an intriguing and rare XX/X0 sex chromosome system, and diploid number of 68 for females and 67 for males [22], and although there is evidence of karyotypic variations between populations, the chromosomal data are limited to the description of the diploid number and pattern of constitutive heterochromatin [22, 23].

Molecular cytogenetics approaches using repetitive sequences, such as 18S and 5S rDNAs and simple short repeats (SSRs or microsatellites), surely provides important information about chromosome evolution and sex chromosome origin [24–27]. Unfortunately, most cytogenetic studies in freshwater stingrays, including *Potamotrygon wallacei*, are scant (only 11 species were karyotyped—see Table 1 of Cruz et al. [28]) and limited to classical techniques (Giemsa staining, C-banding, and Ag-NOR) [22, 23, 29, 30] and only recently, Cruz and colleagues [28] applied molecular tools in their analyses.

In this study, we revisited the karyotype of *P*. *wallacei*, using more refined and combined classical and molecular cytogenetic analyses in order to provide new insights about the karyotypic organization of the species, combining with a paleogeographic modeling to investigate suitable areas for its occurrence and design possible demographic events that led to its current distribution and the karyotypic variations observed between populations.

## Material and methods

Source of specimens, Mitotic chromosome preparation, DNA extraction and Banding procedures.

We analyzed 20 specimens of *P*. *wallacei* from two points of Negro River (Tupé Lake—lower Negro River 1 ♂ and 1 ♀; Anavilhanas—middle/lower Negro River 7 ♂ and 11 ♀). The animal collection occurred under SISBIO permanent license nº 28095–4 issued by Instituto Brasileiro do Meio Ambiente (IBAMA). The individuals were euthanized with the addition of eugenol in the water. The mitotic chromosome preparation was obtained according to Gold et al. [31] from spleen cells. Additionally, total genomic DNA samples (gDNAs) were extracted from muscle tissue with the Wizard^®^ Extraction Kit (Promega) following the manufacturer’s recommendations. For detection of heterochromatin (C-banding) we followed Sumner [32, 33] protocols. Voucher specimens were deposited in the Ichthyological Collection of the National Institute of Amazonian Research (INPA-ICT 059872, 059873, 059874) and the animal handling were performed under license nº 013/2021 (CEUA/INPA).

### Probes labeling

The 18S and 5S rDNA probes were amplified by PCR using the following primers: 18S F (5′-CCG CTT TGG TGA CTC TTG AT-3′) and R (5′-CCG AGG ACC TCA CTA AAC CA-3′) [34], and the primers 5S F (5′-TAC GCC CGA TCT CGT CCG ATC-3′) and R (5′-CAG GCT GGT ATG GCC GTA AGC-3′) [35]. The 5S and 18S rDNA probes were labeled with Spectrum Green-dUTP and Spectrum Orange-dUTP, respectively, by nick translation according to the manufacturer’s recommendations (Roche, Mannheim, Germany).

Oligonucleotide probes containing microsatellite sequences (AC)_15_, (AG)_15_, (GT)_15_, (AT)_15_, (CAT)_15_, (CAC)_15_, (GATA)_4_, (AATC)_15_, and (TTAGGG)_n_ were directly labeled with Cy5 during synthesis by Sigma (St. Louis, MO, USA).

### Fluorescence in situ Hybridization (FISH)

Chromosomal mapping was performed under high stringency conditions on metaphase chromosome spreads of *P*. *wallacei* according to the protocol described by Pinkel et al. [36], for that, 50 μL of the hybridization mixture (2.5 ng/μL probes, 50% deionized formamide, 10% dextran sulfate and 20x SSC) were dropped on the slides, and the hybridization was performed for 24 h at 37 °C in a moist chamber containing distilled water. Post-hybridization washes were made with formamide 15% and 2xSSC Tween 0.5%. The chromosomes were counterstained with DAPI (1.2 μg/mL) and mounted in antifade solution (Vector, Burlingame, CA, USA).

### Microscopic analysis

We analyzed 20 metaphases spreads per individual to confirm the diploid number (2n), C-banding pattern and FISH results. The images were captured using an Olympus BX51 microscope (Olympus Corporation, Ishikawa, Japan) with Cool SNAP, and processed using Image-Pro Plus 4.1 software (Media Cybernetics, Silver Spring, MD, USA). The chromosomes were measured using Image J software and classified according to Levan et al. [37] in metacentric (m), submetracentric (sm), subtelocentric (st) and acrocentric (a).

### Paleogeographic modelling

Potential climatic niche for *P*. *wallacei* was based on occurrence data for 15 localities from the current study, 29 localities from Sistema da Informação sobre a Biodiversidade Brasileira (SiBBr) and 11 localities from Global Biodiversity Information Facility (GBIF). Climate data for current conditions were produced by interpolation of weather stations information from years 1979–2013 and are available in the BioClim—Chelsa Climate [38]. For each occurrence site, 19 bioclimatic variables were extracted from PaleoClim.org Data, with 5 minutes cell resolution [39]. For these variables the variance inflation factor (VIF) was calculated to exclude the highly correlated variables from the set using USDM packet in R software [40].

We used ensembles for 5 different modeling methods: Distance-based model, which assume that species geographic distribution is constrained by climatic tolerances (Bioclimatic Models—BIOCLIM); Regression-based or "statistical" methods that can fit a larger number of parameters to different types of relationships between species occurrence and environmental variables (Generalized Linear Models—GLM, Generalized Additive Models—GAM and species distributions by estimating the probability of occurrence using presence data—MaxLike), and Machine-learning method (Maximum Entropy—MaxEnt), the most complex algorithm in this set, which attempt to maximize the relationship between occurrences and predictors, while minimizing the number of parameters and estimating the probability distribution of maximum entropy (i.e., closest to uniform) subject to the constraint that the expected value of each environmental variable (or its transform and/or interactions) under this estimated distribution matches its empirical average [41].

Calibration of the models was performed with present climatic conditions at 30 arc-seconds resolution. Projections for the Last Interglacial (LIG, ca. 130,000 years ago); Last Glacial Maximum (LGM, ca. 21,000 years ago) and Early, Mid and Late Holocene (ca. 12,000, 8,000, 4,000 years ago, respectively) were carried out at 5 arc-minutes resolution. The data were download from PaleoClim (paleoclim.org) and the resulting maps were displayed using the USDM packet [40] in R (R Development Core Team 2017) (https://cran.rproject.org/web/packages/usdm/index.html). A total of five simulations were performed with each algorithm and only simulations with True Skill Statistic (TSS), Area Under The Curve (AUC) and Receiver Operating Characteristics (ROC) curve higher than 0.8 were kept.

## Results

### Diploid number and heterochromatin patterns

**Fig 1:**
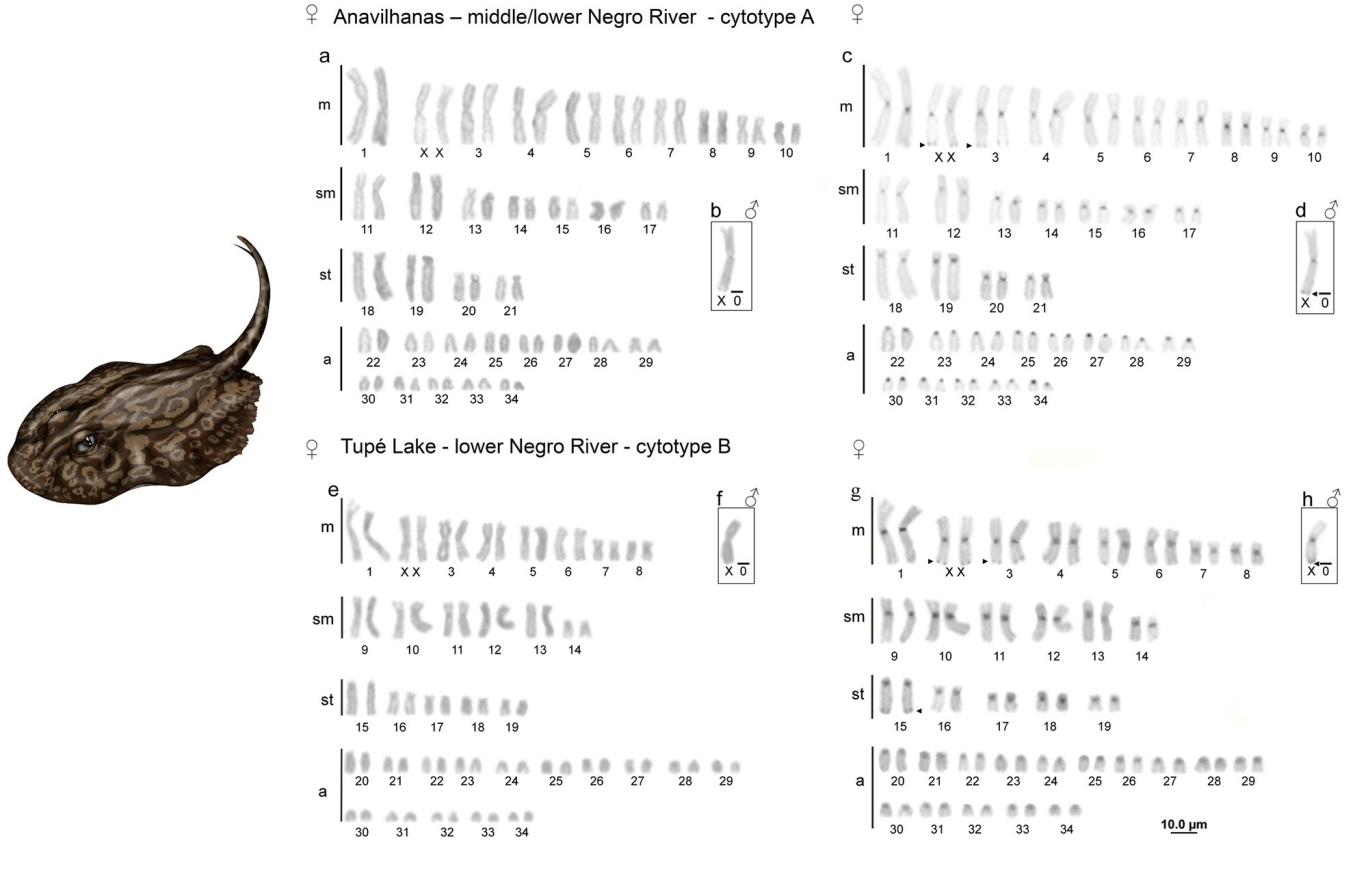
Giemsa and C-banding karyotypes. Giemsa and C-banding karyotypes from Anavilhanas population (a-d)–cytotype A. Giemsa and C-banding karyotypes from Tupé population—cytotype B (e-h) triangles indicating terminal heterochromatic regions. Illustration by Lucas Kías.

All females of *P*. *wallacei* presented 2n = 68 chromosomes whereas the males presented 2n = 67 chromosomes, highlighting the presence of an XX/XO sex chromosome system. We found two cytotypes for *P*. *wallacei* in our analyses, with notable differences in the karyotype formula. The first, named cytotype A (samples from Anavilhanas—middle/lower Negro River) presented males with 19m+14sm+8st+26a and females with 20m+14sm+8st+26a, and the second, named cytotype B (from Tupé Lake—lower Negro River) presented males with 15m+12sm+10st+30a and females 16m+12sm+10st+30a (Fig 1). The heterochromatin was detected in centromeric regions of all chromosomes in both cytotypes, in addition, terminal blocks were found on *q* arms of the third pair and in two X in females and in the X of males in both cytotypes (Fig 1c, 1d, 1g and 1h). Furthermore, the cytotype B also presented a heterochromatic region on the terminal position of *q* arm of the pair 15 (Fig 1g).

### 18S and 5S rDNA chromosomal mapping

**Fig 2:**
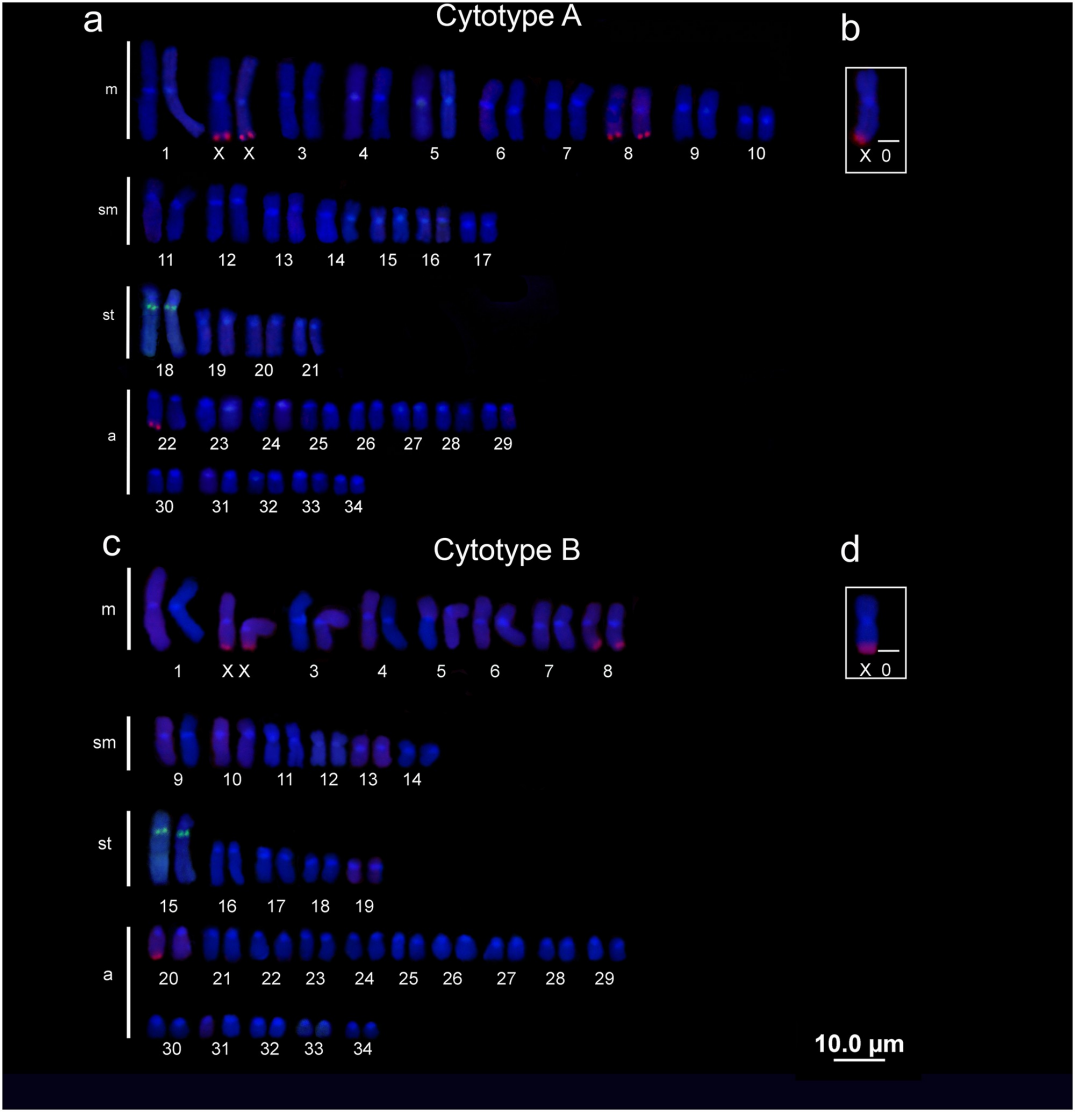
Chromosome mapping of 18S and 5S rDNAs. Chromosome mapping of 18S (red) and 5S (green) rDNAs in cytotype A (a-b) and cytotype B (c-d).

*P*. *wallacei* exhibited 5 sites of 18S rDNA in all females and 4 sites in the males, while the 5S rDNA showed single markings for both sexes. The samples from middle Negro River—cytotype A—showed hybridized signals of 18S rDNA on the terminal position of *q* arm in the XX/X0 chromosomes, 8^th^ pair (m) and in one chromosome of 22^nd^ pair (a), in addition, the 5S sites were found on the *q* arm of 18^th^ pair (the largest subtelocentric pair). On the other hand, the cytotype B showed 18S sites on the terminal regions of *q* arm in the XX/X0 chromosomes, 8^th^ pair (m) and one chromosome of the 20^th^ pair (a), and the 5S rDNA was mapped on the *q* arm of 15^th^ pair (the largest subtelocentric pair). Interestingly, both cytotypes presented 18S rDNA sites in two bi-armed pairs and one uni-armed chromosome (Fig 2).

### Microsatellite and Telomeric (TTAGGGn) mapping

**Fig 3:**
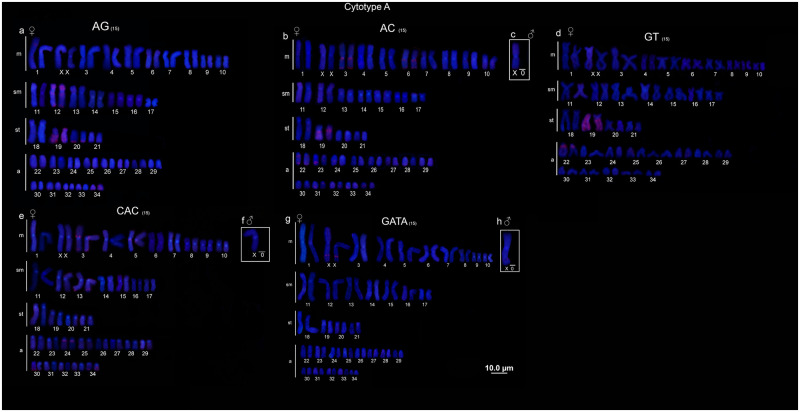
Microsatellites cytotype A. Microsatellite mapping (a) (AG)_15_, (b) (AC)_15_, (c) (GT)_15_, (d) (CAC)_15_ and (e) (GATA)_4_ in metaphases of cytotype A.

**Fig 4:**
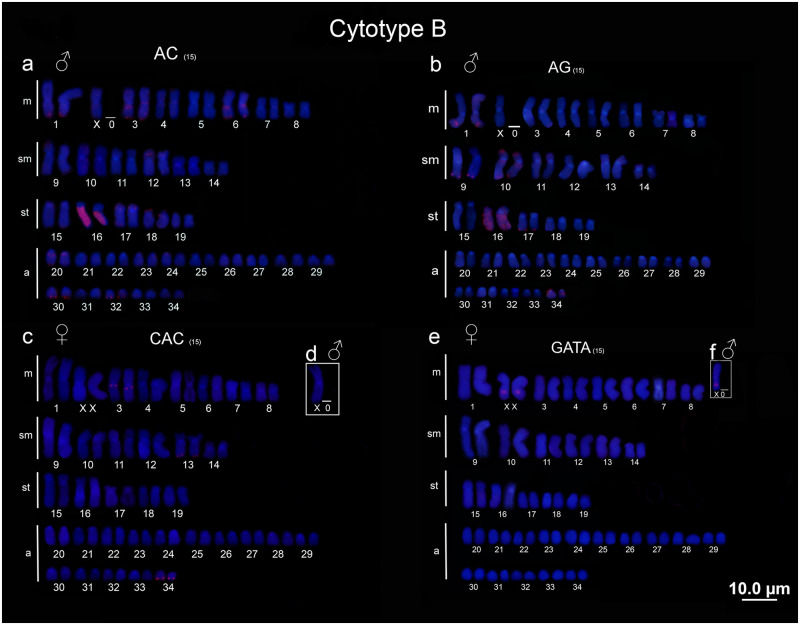
Microsatellites cytotype B. Microsatellite mapping (a) (AG)_15_, (b) (AC)_15_, (c) (CAC)_15_ and (d) (GATA)_4_ in metaphases of cytotype B.

Conspicuous hybridization signals for (AC)_15_ and (AG)_15_ were observed in the 19^th^ pair in the cytotype A and 16^th^ pair in the cytotype B (the first subtelocentric pair in both cases), interestingly, for both cytotypes the homologous showed different patterns, one chromosome was completely hybridized whereas the other showed markers only on the *q* arm (Figs 3a and 3b, 4a and 4b). In addition, cytotype B also presented (AC)_15_ clusters in the pericentromeric and terminal position of *q* arm (3, 6, and 20 pairs), terminal region of *p* arm (12, 17 and 18 pairs), and terminal position of *q* arm (1, 30 and 32 pairs) (Fig 4a). On the other hand, (AC)_15_ clusters were observed in pericentromeric and terminal regions of *q* arm (3, 6 and 22 pairs), interstitial sites in the *q* arm (23 and 34 pairs) and terminal position of *q* arm (31 and 33 pairs) for cytotype A (Fig 3b). Besides that, the population from middle Negro River showed amplification of (GT)_15_ SSRs in two chromosome pairs (19^th^ and 22^nd^) (Fig 3d), which was not observed in the population from lower Negro River, once the GT SSRs did not show hybridized signals.

Microsatellite clusters of (CAC)_15_ also showed differences between populations of *P*. *wallacei*. The cytotype A presented pericentromeric and terminal signals in the 3^rd^ and 34^th^ pairs, terminal clusters on the *q* arm of 13^th^ and 30^th^ pairs, and pericentromeric markers in the 5^th^ and 24^th^ pairs (Fig 3e). Besides, the cytotype B presented (CAC)_15_ clusters only in three chromosome pairs, pericentromeric in the 3^rd^ pair, and terminal signals on the *q* arm in the 13^th^ and 34^th^ pairs (Fig 4c). Finally, the (GATA)_4_ clusters showed association with the XX/X0 sex chromosome system in both populations. The males presented one site, while the females presented two sites of this sequence (Figs 3g and 4e). Interestingly, no amplification of (AT)_15_, (CAT)_15_ and (AATC)_15_ has been detected in both populations. Telomeric probes hybridized in terminal position of all chromosome pairs with no traits of interstitial telomeric sites (ITS) (S1 Fig).

### Paleogeographic modelling

**Fig 5:**
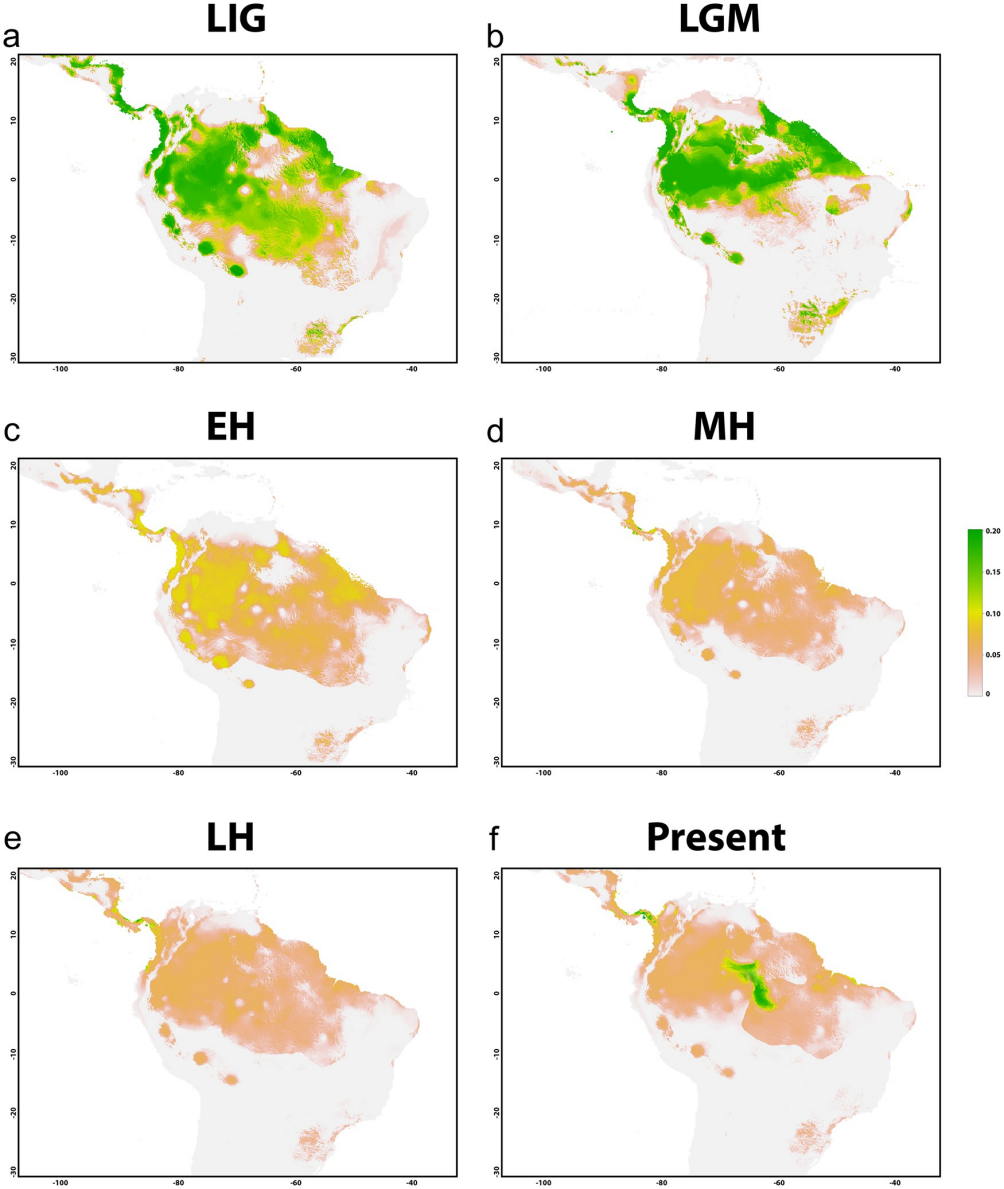
Paleogeographic modelling. Potential climatic niche for *P*. *wallacei* recovered in our analyses. (a) Last Interglacial (LIG), (b) Last Glacial Maximum (LGM), (c) Early Holocene (EH), (d) Middle Holocene (MH), (e) Late Holocene (LH) and Present (f). Intense green indicates areas that are more susceptible to the occurrence of the species and yellow tones indicate areas that are less susceptible for *P*. *wallacei*. The data were download from PaleoClim (paleoclim.org) and the resulting maps were displayed using the USDM packet in R (R Development Core Team 2017) (https://cran.rproject.org/web/packages/usdm/index.html).

Our modelling generated a distribution for the Present that is highly concordant with registered occurrences, basically restricted to the Negro River (Fig 5), a much smaller area than observed in the Last Interglacial (LIG) and Last Glacial Maximum (LGM), where the likely distribution of *P*. *wallacei* was far more representative, with suitable areas located from east to west across the north of South America, predominantly in the regions that matches with the Amazon and some other regions (Fig 5).

The Early and Middle Holocene on the other hand, were the periods that showed less suitable areas for the occurrence of the species, presenting lesser marginally stable locations among Amazon and northern regions of South America (Fig 5). However, in the Late Holocene some small suitable areas for occurrence of *P*. *wallacei* were retrieved for some regions that corresponds to Colombia and Panamá, a pattern quite similar to that recovered for the Present projections (Fig 5).

## Discussion

Cytogenetic studies on cartilaginous fishes conducted have shown peculiarities like karyotype characterized by high diploid number and heterochromatin blocks only on the centromeric region [42–46]. Besides that, on Potamotrygoninae the data available shows a reduction on the diploid number probably mediated by centric fusions which increases the number of bi-armed chromosomes and reduced the number of acrocentric chromosomes [22, 23, 28, 30].

The 2n of *Potamotrygon* species range from 64 to 67/68 chromosomes (most karyotyped species presented 2n = 66), these evidences support the idea that centric fusions shaped the karyotype of this genus increasing the number of bi-armed chromosomes [22, 23, 28, 30, present study]. Valentim et al. [22] described the karyotype of *P*. *wallacei* (cited as *Potamotrygon* sp. C) and latter Valentim et al. [23] reported a polymorphism in specimens from Negro River (near to Barcelos city). In our analyses we found two different karyotypes that we call cytotype A from middle Negro River (20m+14sm+8st+26a) and cytotype B from lower Negro River (near to Manaus) (16m+12sm+10st+30a). Overall, our data is different from those previously reported by Valentim et al. [22, 23], and taken together, those findings could be evidence that pericentric inversions are acting in the karyotype of *P*. *wallacei* leading to intra and interpopulation differences on the chromosomal macrostructure [22, 23, present study].

The presence of heterochromatic blocks in centromeric regions are very common, especially in cartilaginous fishes, and are often associated with a structural role displayed by heterochromatin in genome architecture [42–44, 46, 47]. This pattern is commonly found in bone fishes [48–50], and in all freshwater stingrays species cytogenetically characterized [22, 23, 29]. The exception is *P*. *wallacei* here analyzed, whose chromosomes exhibited terminal blocks on the long arms of pairs 2 and 3, a pattern, although relatively uncommon, already seen in some marine species [45].

Another useful tool for chromosomal evolution and organization studies is the mapping of rDNAs which provides valuable insights in many taxa [26, 27, 50–53]. These repetitive sequences have unique evolutionary dynamic rates and fundamental functions, from control the cell maintenance of genomics integrity to shaping sex chromosomes, functions beyond the ribosomal synthesis [54]. Nevertheless, we have only two studies that provided molecular cytogenetics data about freshwater stingrays [28, present study], thus the lack of information remains. For *P*. *wallacei* the pattern found for 18S rDNA (5 sites in females and 4 in males) showed a trend to the decrease of sites for this sequence in the freshwater stingrays comparing to the marine ones [44, 46]. Besides that, our findings differ from Cruz et al. [28] once the authors reported sites of this sequence in metacentric and acrocentric chromosome pairs (4 chromosome pairs) and here, we found this rDNA sequence in two bi-armed chromosomal pairs and in only one acrocentric chromosome (pattern shared between both cytotypes). In addition, while in the marine species the 18S rDNA was found in centromeric and terminal positions (in some cases in both chromosome arms) [44, 46] in freshwater stingrays the sites are only found on the terminal position of long arm [28, present study]. This variations could be a result of centric fusions, deletions or even translocations involving 18S sites during the karyotypic evolution in freshwater stingrays [28, present study].

On the other hand, the 5S rDNA seems to be conserved among Potamotrygoninae once we found the same number of sites in the same chromosome pair reported for *Potamotrygon motoro* Müller & Henle, 1841 and *Potamotrygon* sp. (interstitial in the first subtelocentric pair) [28] which might be a homeologous chromosome pair for these species of freshwater stingrays. In marine species two patterns were found for cartilaginous fishes, *Raja montagi* Fowler, 1910 and *Taeniura lymma* Forsskal, 1775 with 2 pairs carrying this sequence [44], and only one uni-armed chromosome pair in *Torpedo* species [46]. Nevertheless, further investigation is needed to establish the evolutionary trends of rDNAs in freshwater stingrays. Thus, although these markers (5S and 18S rDNAs) have been detected in different chromosomal pairs in the two populations of *P*. *wallacei* analyzed here, we believe that these pairs present homeology and the variation found is due to the action of chromosomal rearrangements that acted independently among them.

The association between repetitive sequences, such as rDNAs and microsatellite sequences (short sequences repeats—SSRs), and sex chromosomes is a common trace among several neotropical fishes [26, 51, 53, 55, 56]. *Potamotrygon wallacei* also followed this evolutionary path showing association of 18S rDNA sites and (GATA)_4_ motifs with its XX/X0 sex chromosome system, suggesting that these sequences may have played a role in the differentiation of this unique sex chromosome system.

Microsatellites sequences are highly polymorphic at both species and population scales [57, 58]. These sequences are often associated with regulatory function in the genome architecture, structural organization of DNA, chromatin organization and gene activity [59]. SSRs are also associated with sex chromosomes in many taxa [24, 25, 51]. In our analyses, we also found differences in the distribution pattern of the SSRs used. Curiously (AC)_15_ and (AG)_15_ showed strong association with one chromosome pair, the 16^th^ pair in the cytotype B (lower Negro River) and the 19^th^ pair in the cytotype A (middle Negro River). Thus, we hypothesized that these chromosome pairs are homeologous, since they share the same pattern of distribution for this sequence, even with differences between homologous, and in both cytotypes they are the second subtelocentric pair (Figs 3a and 3b; 4a and 4b).

These differences between the homologous may be a result of the dynamic behavior of these sequences, together with its expansion and contraction in the genomes due to slippage during DNA replication and high mutation rates [57, 58, 60]. Those characteristics make SSRs a useful tool to investigate chromosomal variations in closely related species or even in different populations [24, 57, 59]. Moreover, the differences in the patterns of SSRs (especially the (GT)_15_ detected solely in one population) could be related to different landscapes impacting the distribution of these sequences in *P*. *wallacei*, thus SSRs might provide important information about chromosomal rearrangements in different populations.

Although the association of SSRs motifs with sex chromosomes has been documented in many vertebrate groups [24, 25, 27, 51, 61, 62], unfortunately, data about the distribution of SSRs in Potamotrygoninae species are limited to the present study. While most of SSRs used here showed amplification in several chromosomes, (GATA)_4_ motif was associated with the X chromosome in *P*. *wallacei* (two sites in the female and one in males). (GATA)_4_ motif is the main component of satellite DNA isolated from the W chromosome of the snake *Elaphe radiate*, being associated with sex chromosome evolution in several eukaryotic species [63–65]. Likewise, this sequence was found in the XX/XY sex chromosomes of *Hoplias malabaricus*, although no significant differences were found between its distribution on the X or Y [27]. On the other hand, Viana et al. [24] reported association between (GATA)_4_ motif and X chromosomes in turtle *Chelus orinocensis* Vargas-Ramírez, 2020 and *Chelus frimbriata* Schneider, 1783, in spite of other closely related species have shown a different pattern [25]. Thus, taken together, our findings suggest that this sequence indeed played a role in the differentiation of the sex chromosomes of *P*. *wallacei*.

Although the association between sex chromosomes and (AG)_15_ and (AC)_15_ motifs have been reported in some bone fish [61, 62], our data follow the general pattern found in other groups, showing pericentromeric and terminal clusters in autosomal chromosomes [50, 62, 66]. In this case, these microsatellite clusters might play a structural role and the pattern found here must be a result of different pressures that shaped the distribution of these SSRs in the karyotype in the *P*. *wallacei*.

Our experiments did not show any ITS signals despite the differences in the karyotypic formula, this absence of ITS might be caused by successive losses and degeneration, leading to gradual shortening of the nonfunctional telomeric arrays as seen in other animal groups [49, 50, 67, 68]. However, the differences in the karyotypic formula might indicate that inversions play a key role in the karyotype differentiation of *P*. *wallacei* populations. Similarly, Cruz et al. [30] reported chromosomal variations related to the karyotypic formula and Ag-NOR sites in *Potamotrygon falkneri* Castex & Maciel, 1963 from different locations. These differences reinforce that, in addition to fusions, other chromosomal rearrangements are acting in the karyotypic diversification of freshwater stingrays. Additionally, since the lower Amazon was colonized by linages from Upper Amazon regions [69], the karyotype diversity in this group might be a result of these past migration movements.

The potential climatic niches recovered in our projections revealed an area much larger than the current distribution of the species, mainly during the LIG (130 Ma) and LGM (21 Ma). However, the most probably origin of freshwater stingrays was at 26 Ma and the first representatives may occupied the Upper Amazon regions and then colonized other drainages, as Negro/Branco [69]. In addition, our analyses showed changes in this scenario in the EH, as in MH and LH, at this point the region was passing through several changes in the basin conformation, as well as, in the same period the most intense uplifts of Andes occurred, which strongly affected the dynamic of the Amazon basin and consequently the entire ichthyofauna of the region [70]. The rivers that previously drained towards the Pebas system, at this point began to flow towards the Atlantic [70–72] and this led to migratory movements from the Upper Amazon to the Brazilian shield regions and from Upper Amazon to lower Amazon [69]. These intense environmental changes together with the populational dynamics changes probably affected *P*. *wallacei* distribution and led to the fixation of different chromosomal rearrangements in current populations.

In the last decade, a few studies with classical cytogenetics approach were performed with freshwater stingrays’ species and provided information about the general trends of chromosome evolution in this group. Our study is the first to offer extensive molecular cytogenetics data about the endemic *Potamotrygon wallacei*, especially regarding the mapping of SSRs motifs and rDNAs. Our data surely brings important information on the role of the chromosomal inversions that shaped the karyotype formula and promoted the changes observed in the chromosomal macrostructure in stingrays. The present study is the first of a series involving classical and molecular investigation in freshwater stingrays in order to provide insights about chromosomal evolution and sex chromosome systems origin.

## Supporting information

S1 Fig Telomeric (TTAGGGn) mapping. *P*. *wallacei* metaphases after (TTAGGG)n probe hybridization (red) showing absence of ITS sites. (a) cytotype A and (b) cytotype B.
(TIF)
