# Supplementary figures and images for: Karyotypic variation of two populations of the small freshwater stingray Potamotrygon wallacei Carvalho, Rosa & Araújo 2016: A classical and molecular approach
Source: PLoS One. 2023 Jan 20;18(1):e0278828. doi: 10.1371/journal.pone.0278828 (PMC9858463; doi:10.1371/journal.pone.0278828)

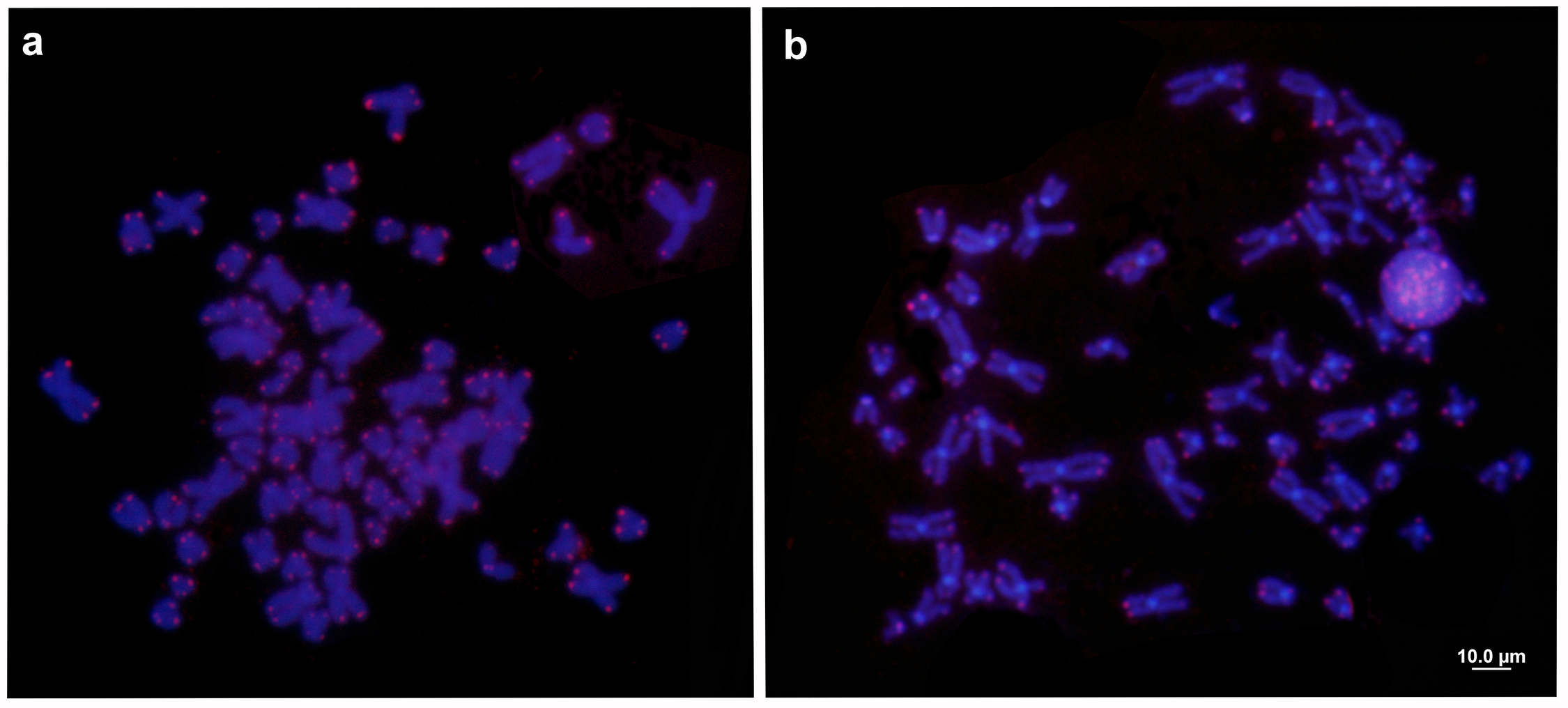

Supplement: S1 Fig — P. wallacei metaphases after (TTAGGG)n probe hybridization (red) showing absence of ITS sites. (a) cytotype A and (b) cytotype B. (TIF) [file pone.0278828.s001.tif]
